# Synthesis and radiolabeling of a polar [^125^I]I‐1,2,4,5‐tetrazine

**DOI:** 10.1002/jlcr.4009

**Published:** 2022-12-30

**Authors:** Natasha Bidesi, Vladimir Shalgunov, Umberto Maria Battisti, Lars Hvass, Jesper Tranekjær Jørgensen, Christian B. M. Poulie, Andreas I. Jensen, Andreas Kjaer, Matthias M. Herth

**Affiliations:** ^1^ Department of Drug Design and Pharmacology, Faculty of Health and Medical Sciences University of Copenhagen Copenhagen Denmark; ^2^ Department of Clinical Physiology, Nuclear Medicine and PET Rigshospitalet Copenhagen Denmark; ^3^ Department of Clinical Physiology, Nuclear Medicine and PET & Cluster for Molecular Imaging, Copenhagen University Hospital – Rigshospitalet & Department of Biomedical Sciences University of Copenhagen Copenhagen Denmark; ^4^ Center for Nuclear Technologies (DTU Nutech) Technical University of Denmark (DTU) Roskilde Denmark

**Keywords:** bioorthogonal, click chemistry, idodine‐125, pretargeting, tetrazine

## Abstract

Pretargeting imaging has gained a lot of prominence, due to its excellent bioorthogonality and improved imaging contrast compared to conventional imaging. A new iodo tetrazine (Tz) derivative has been synthesized and further developed into the corresponding iodine‐125 (^125^I) analog (**12**), via the trimethylstannane precursor. Radiolabeling with either *N*‐chlorosuccinimide or chloramine‐T, in either MeCN or MeOH proceeded with a radiochemical conversion (RCC) of >80%. Subsequent deprotection only proved successful, among the tested conditions, when the radiolabeled Tz was stirred in 6‐M HCl_(aq.)_ at 60°C for 2.5 h. To the best of our knowledge, this is the first H‐tetrazine labeled with iodine. In vivo investigations on the pretargeting ability of **12** are currently under way.

## Graphical abstract

A new iodo tetrazine suitable for in vivo pretargeting has been synthesized and further developed into the corresponding iodine‐125 analogue. The compound was obtained via trimethylstannane precursor with N‐chlorosuccinimide or chloramine‐T followed by deprotection in acidic media with a radiochemical conversion of 42%. In vivo investigations on its pretargeting ability are currently under way.

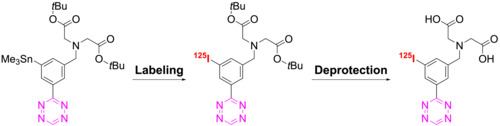

## 1 INTRODUCTION

**FIGURE 1:**
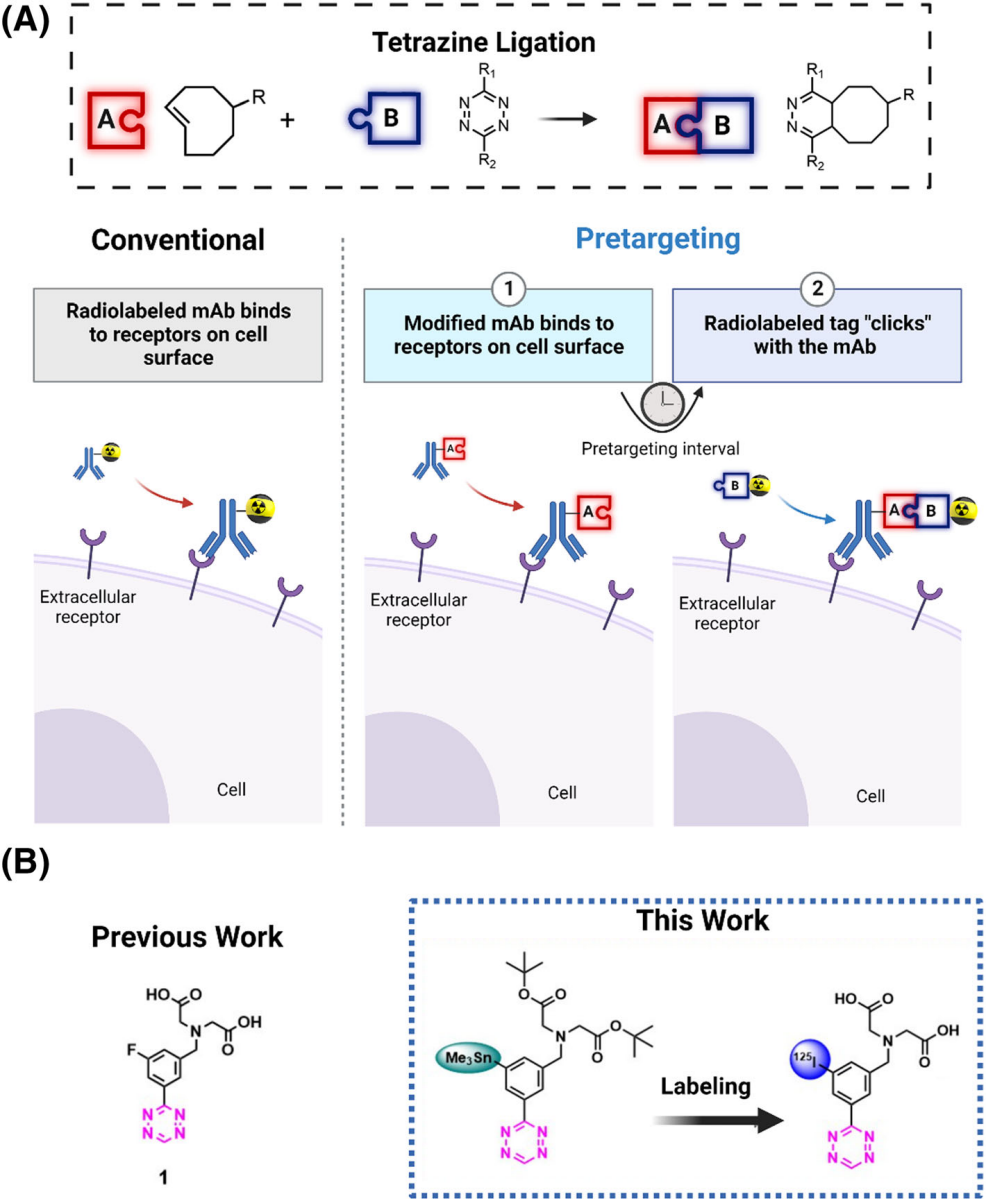
(A) Conventional immunoimaging vs. pretargeting immunoimaging based on the tetrazine ligation.^3^ (B) Strategy to develop a [^125^I]I‐Tz suitable for in vivo pretargeting chemistry. Our approach was based on compound **1**, recently reported successful pretargeted imaging agent.^6^

Monoclonal antibodies (mAbs) have emerged as an important tool in the diagnosis and treatment of multiple diseases such as cancer, due to their excellent target selectivity and high binding affinities.^1^, ^2^ One of the major drawback of mAbs are their pharmacokinetic properties, such as slow target accumulation and slow blood clearance.^3^, ^4^ This is why, for the use of these vectors, radionuclides with longer half‐lives are required to match their pharmacokinetic profile.^3^ Consequently, patients are undergoing an unnecessary radiation burden and sometimes even at a magnitude prohibitive for clinical studies.^5^ An innovative concept for avoiding previous mentioned limitations is pretargeting, because in this concept the accumulation of mAbs is not directly influencing the imaging process anymore (Figure 1A). Due to the previously mentioned properties, mAbs are often used in pretargeting strategies and can result in high target‐to‐background ratios with low nondisplaceable binding, which in the context of nuclear imaging makes them ideal pretargeting vectors.

In pretargeting, the mAb is modified with a chemical tag, which can react bioorthogonally with a small molecular imaging agent. The modified mAb is typically allowed to circulate for several days before the administration of the imaging agent. This two‐step procedure enables for the use of shorter lived radionuclides, decreasing the overall radiation burden to the patient. Currently, the most auspicious example of this biorthogonal reaction is the tetrazine‐ligation, which is an inverse‐electron demand Diels–Alder (IEDDA) reaction between a 1,2,4,5‐tetrazine (Tz) and the *trans*‐cyclooctene (TCO). Multiple examples with different nuclides are reported indicating the potential of this ligation.^7^, ^8^, ^9^, ^10^, ^11^, ^12^, ^13^, ^14^, ^15^, ^16^, ^17^, ^18^, ^19^, ^20^, ^21^, ^22^, ^23^

Radioiodination of biomolecules is commonly carried out in the study of a compound's pharmacokinetics and pharmacodynamic profile, due to their physical properties and the already established iodine/iodide chemistry. For these reasons, iodine is often used for developing tracers such as small molecules and proteins, and to lesser extent peptides.^24^ Specifically the radioisotope iodine‐125 (^125^I) can allow a fast and cheap quantitative biodistribution of the mAb by radioactive counting of the low energy gamma emission (35 keV). Furthermore, the iodine radioisotopes form theranostic pairs (^123^I, ^124^I, ^125^I, and ^131^I), which makes them a convenient tool to develop theranostic agents.^25^, ^26^, ^27^

Recently, our group reported the labeling of highly reactive polar tetrazines with fluorine‐18 (^18^F). These compounds were radiolabeled in good radiochemical yield (RCY) and radiochemical purity (RCP), and in particular, compound **1** (Figure 1B) displayed promising data in an in vivo pretargeting experiment.^6^

In this study, we describe the synthesis and the first radiolabeling of its ^125^I analog. This compound could potentially be useful for pretargeted strategies. The tracer was obtained from a trimethylstannane precursor with a one‐pot two‐step procedure. This the first time that the labeling of an H‐Tz with iodine has been reported. Moreover, the results obtained for this could be translated in the future to more clinically relevant nuclides. As a matter of fact, ^125^I represents a cheap and affordable surrogate for both iodine‐131 (^131^I) and astatine‐211 (^211^At) due to a comparable size and similar labeling procedures.

## 2 RESULTS AND DISCUSSION

### 2.1 Synthesis of Tz derivatives

**SCHEME 1:**
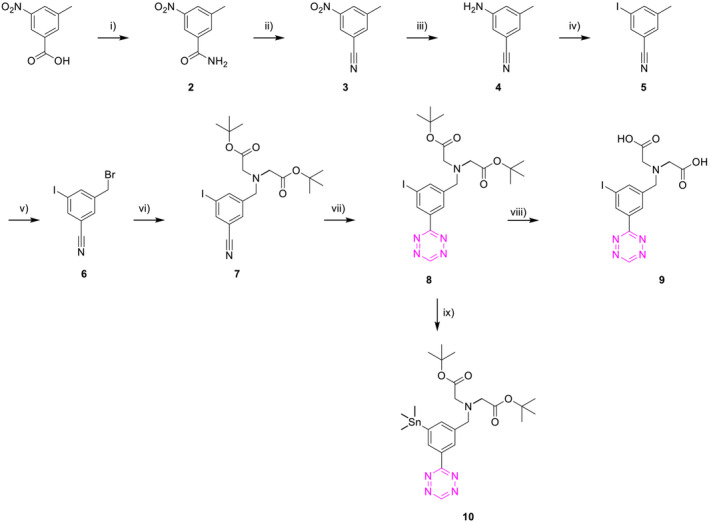
Synthesis of H‐Tzs and their precursors. *Reagents and conditions*: (i) (a) SOCl_2_, reflux, 2 h; (b) NH_3_, H_2_O, THF, 0°C to rt, 30 min; 93%; (ii) triflic anhydride, Et_3_N, CH_2_Cl_2_, 0°C to rt, 30 min; 92%; (iii) iron powder, AcOH, MeOH, reflux, 2 h, 98%; (iv) NaNO_2_, HCl, KI, acetone, H_2_O, 0°C to reflux, 2 h, 76%; (v) NBS, AIBN, MeCN, reflux, 24 h, 49%; (vi) di‐*tert*‐butyl iminodiacetate, K_2_CO_3_, MeCN, 25°C, 24 h, 89%; (vii) (a) CH_2_Cl_2_, S_8_, NH_2_NH_2_·H_2_O, EtOH, 50°C, 24 h, (b) NaNO_2_, AcOH, rt, 30 min, 26–27%; (viii) TFA, CH_2_Cl_2_, 25°C, 2 h, 62–65%; (ix) (Me_3_Sn)_2_, Pd (PPh_3_)_4_, THF, 65°C, MW, 3 h, 47–66%

The selected Tzs were obtained similarly to what was recently reported (Scheme 1).^6^, ^28^ 3‐Methyl‐5‐nitrobenzoic acid was converted into the corresponding amide (**2**) and was subsequently dehydrated to give nitrile **3**. The nitro group on **3** was reduced with acetic acid/iron, to yield aniline **4**, which was converted to the corresponding iodo‐analog **5**, through a Sandmeyer reaction. Radical bromination and alkylation of di‐*tert*‐butyl iminodiacetate gave nitrile **7**. The latter was converted into the corresponding H‐Tzs **8** employing the methodology published by Qu et al.^29^ Deprotection with TFA followed by preparative HPLC afforded the reference compound **9**. Finally, the trimethylstannane precursor **10** was obtained through a Pd‐catalyzed cross‐coupling reaction from the corresponding iodine derivative.

### 2.2 Labeling of [^125^I]I‐Tz derivatives

**FIGURE 2:**
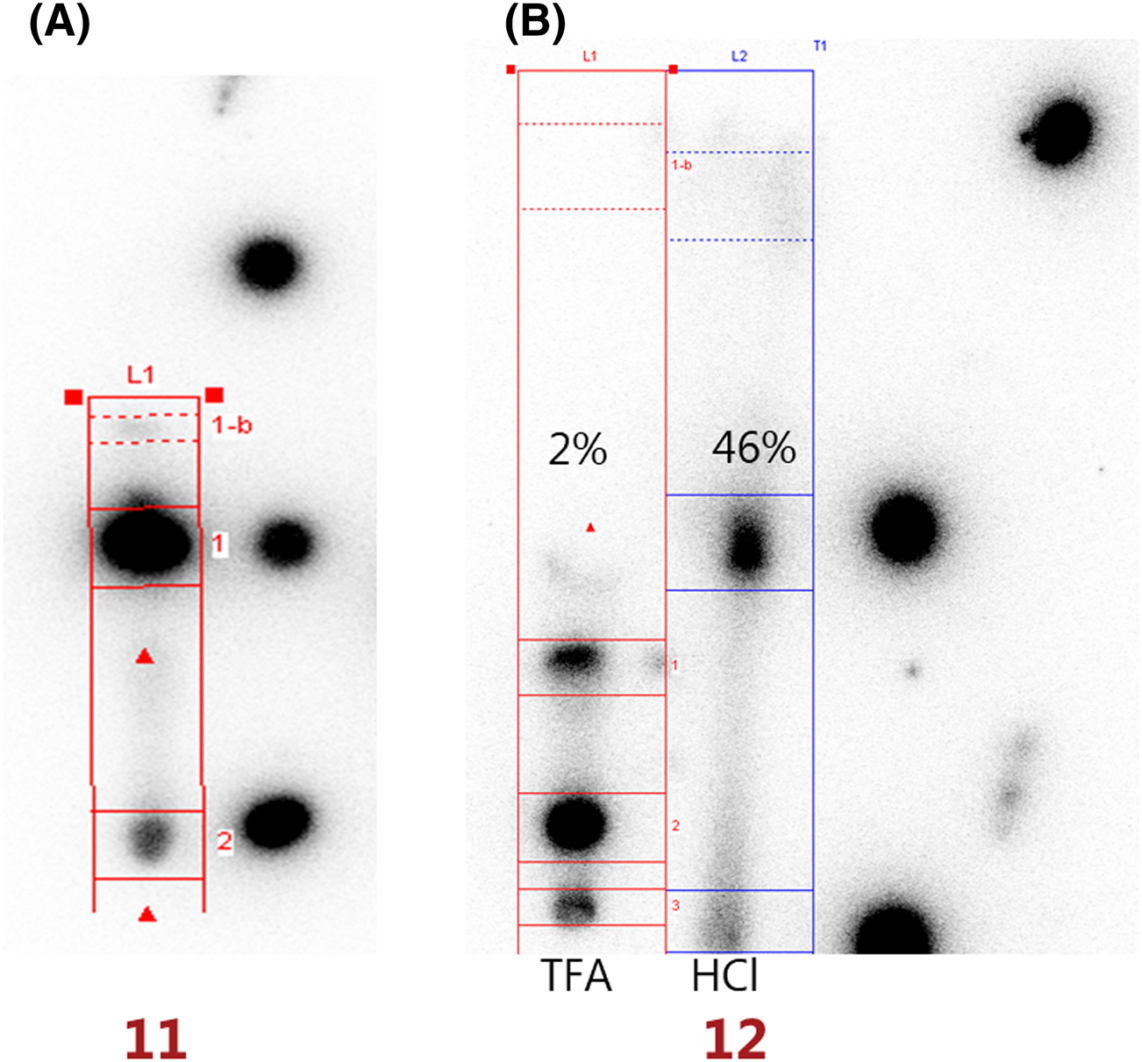
2A: ^125^I‐iodination of **10**, RadioTLC (NP Hept/EtOAc 8:2, v/v): Left lane: crude reaction mixture **11**. Right lane: R_*f*_ = 0, cold reference and R_*f*_ = 1 marked with Na[^125^I]I. 2B: Deprotection. RadioTLC (RP MeCN/H_2_O, 1:2, v/v). Leftt lane: crude reaction mixture **12** with TFA, at 60°C, used for deprotection. Middle lane: crude reaction mixture **13** with 6 M HCl_(aq.)_, at 60°C, used for deprotection. Right lane: R_*f*_ = 0, cold reference and R_*f*_ = 1 marked with Na[^125^I]I.

A modified procedure of Albu et al. was used for radioiodination of **10**.^30^ Radiohalodestannylation followed by deprotection was achieved in a one‐pot two‐step sequence resulting in solutions of 1 MBq/ml. Multiple conditions were screened as depicted in Table 1. For the radioiodination, the two solvents investigated did not result in significant variation in radiochemical conversion* (RCC). Regarding the deprotection step, the best condition proved to be 6‐M HCl_(aq.)_ at 60°C for 2.5 h. Higher temperatures were not evaluated due to the known instability of the H‐tetrazine core. The optimized and scaled up reaction (starting from 20 MBq/ml) resulted in an overall RCC of 46 ± 31% (*n* = 5) and upon semipreparative HPLC purification and formulation on a SepPak C18 cartridge, an RCY of 5% (ND = 3; SD = ±1%). was obtained; **12** was stable in PBS/EtOH (95/5) for at least 48 h and was able to react instantaneously with TCO‐PNB ester, which was determined by radio‐TLC (Figure 2).

**TABLE 1:** Optimization of reaction conditions of the two‐step one pot system used. RCC*: Intermediate radiochemical conversion of the iodination, measured by an aliquot of the reaction mixture. Values obtained with *n* = 5 reporting standard deviation. RCC: overall conversion over two steps

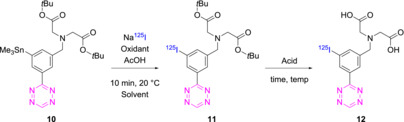		
Labeling		
Solvent	Oxidant	RCC* (%)
MeCN	CAT	87 ± 2
MeCN	NCS	80 ± 1
MeOH	CAT	82 ± 2
MeOH	NCS	75 ± 12

Deprotection			
Acid	Time (h)	Temperature (°C)	RCC (%)
TFA (neat)	3	20	0
TFA (neat)	2.5	60	0
TFA (neat)	2.5	60	2
6‐M HCl_(aq)_	3	20	0
6‐M HCl_(aq)_	2	60	15 ± 11% (*n* = 5)
6‐M HCl_(aq)_	2.5	60	42 ± 31% (*n* = 5)

## 3 CONCLUSIONS

In summary, we have synthesized a new iodo‐Tz derivative and its corresponding [^125^I]I‐analog. The radiolabeling was performed from the trimethylstannyl precursors with either *N*‐chlorosuccinimide (NCS) or Chloramine‐T (CAT), in either MeCN or MeOH yielded a RCC of >80%. Subsequent deprotection of both *t*Bu‐ester only proved successful when the radiolabeled Tz was stirred in be 6‐M HCl_(aq.)_ at 60°C for 2.5 h. Future investigations on the pretargeting ability of the compound are currently under way.

## 4 EXPERIMENTAL

### 4.1 General

All reagents and solvents were purchased from commercial suppliers and used without further purification. Anhydrous tetrahydrofuran (THF) was obtained from an SG Water solvent purification system (Pure Process Technology). Anhydrous dimethyl sulfoxide (DMSO), *N*,*N*‐dimethylacetamide (DMA), MeCN, and pyridine were purchased from commercially suppliers and stored under argon. Reactions requiring anhydrous conditions were carried out under inert atmosphere (nitrogen or argon) and using oven‐dried glassware (152°C). Syringes used to transfer anhydrous solvents or reagents were purged with argon prior to use. Other solvents were analytical or HPLC grade and were used as received. NMR spectra were acquired on a 600‐MHz Bruker Avance III HD (600 MHz for ^1^H and 151 MHz for ^13^C), a 400‐MHz Bruker Avance II (400 MHz for ^1^H, 376 MHz for ^19^F and 101 MHz for ^13^C), and a 400‐MHz Bruker Avance UltraShield (400 MHz for ^1^H, 376 MHz for ^19^F and 101 MHz for ^13^C), using Chloroform*‐d*, MeOD or DMSO*‐d*_6_ as deuterated solvent and with the residual solvent as the internal reference. For all NMR experiences, the deuterated solvent signal was used as the internal lock. Coupling constants (*J* values) are given in Hertz (Hz). Multiplicities of ^1^H NMR signals are reported as follows: s, singlet; d, doublet; dd, doublet of doublets; ddd, doublet of doublets of doublets; dt, doublet of triplets; t, triplet; q, quartet; m, multiplet; br, broad signal. NMR spectra of all compounds are reprocessed in MestReNova software (version 12.0.22023) from original FID's files. Yields refer to isolated compounds estimated to be >90% pure as determined by ^1^H NMR (25°C) and analytical HPLC. Analytical HPLC method: Thermo Fisher UltiMate 3000 with a C‐18 column (Luna 5 μm C18 (2) 100 Å, 150 mm × 4.6 mm). Eluents: A, H_2_O with 0.1% TFA; B, MeCN with 0.1% TFA. Gradient from 100% A to 100% B over 12 min, back to 100% A over 3 min, flow rate 2 ml/min. Detection by UV absorption at *λ* = 254 nm on a UVD 170 U detector. Thin‐layer chromatography (TLC) was carried out on silica gel 60 F_254_ plates from Merck (Germany). Visualization was accomplished by UV lamp (254 nm). Preparative HPLC was carried out on an UltiMate HPLC system (Thermo Scientific) consisting of an LPG‐3200BX pump (10 ml/min), a Rheodyne 9725i injector, a 10‐ml loop, a MWD‐3000SD detector (254 nm), and an AFC‐3000SD automated fraction collector, using a Gemini‐NX C18 column (21.2 × 250 mm, 5 μm, 110 Å) (Phenomenex) equipped with a guard. Purifications were performed using linear gradients of 0.1% TFA in MilliQ‐H_2_O (A) and 0.1% TFA, 10% MilliQ‐H_2_O in MeCN (B). Data were acquired and processed using Chromeleon Software v. 6.80. Semipreparative HPLC was performed on the same system using a Luna 5μ C18 column (250 × 10 mm) with a flow rate of 3 ml/min. Automated flash column chromatography was performed on a CombiFlash NextGen 300+ system supplied by TeleDyne ISCO, equipped with RediSep silica packed columns. Detection of the compounds was carried out by means of a UV‐vis variable wavelength detector operating from 200 to 800 nm and by evaporative light scattering detector (ELSD). Solvent systems for separation were particular for each compound but consisted of various mixtures of heptane, EtOAc, CH_2_Cl_2_, and MeOH. Microwave‐assisted synthesis was carried out in a Biotage Initiator apparatus operating in single mode; the microwave cavity producing controlled irradiation at 2.45 GHz (Biotage AB, Uppsala, Sweden). The reactions were run in sealed vessels. These experiments were performed by employing magnetic stirring and a fixed hold time using variable power to reach (during 1–2 min) and then maintain the desired temperature in the vessel for the programmed time period. The temperature was monitored by an IR sensor focused on a point on the reactor vial glass. The IR sensor was calibrated to internal solution reaction temperature by the manufacturer. Mass spectra analysis was completed using MS‐Acquity‐A: Waters Acquity UPLC with QDa‐detector. High‐resolution mass spectrometry (HRMS) was performed as matrix‐assisted laser desorption/ionization time‐of‐flight mass spectrometry (MALDI‐TOF). Analyses were performed in positive ion mode with ionization on a ThermoQExactive Orbitrap mass spectrometer (Thermo Scientific) equipped with an AP‐SMALDI 10 ion source (TransmitMIT) and operated with mass resolving power 140,000 at *m/z* 200 and lock‐mass for internal mass calibration. Samples were dissolved in a matrix consisting of 2,5‐dihydrooxybenzoic acid, 15 mg/ml (positive mode).

### 4.2 Organic chemistry

#### 4.2.1 3‐Methyl‐5‐nitrobenzamide (**2**)

3‐Methyl‐5‐nitrobenzoic acid (4.5 g, 24.84 mmol) was refluxed in thionyl chloride (20 ml) for 4 h. The volatiles were removed under reduced pressure and the residue was solubilized in THF (10 ml). The latter was added dropwise to a 28% NH_3_ solution in water (40 ml) at 0°C. The mixture was stirred at room temperature for 30 min. The resulting suspension was filtered to afford 4.18 g (93%) of **2** as a white solid. R_*f*_ = 0.14 (70/30 n‐heptane/EtOAc); ^1^H NMR (600 MHz, DMSO) δ 8.50 (t, *J* = 1.9 Hz, 1H), 8.28 (s, 1H), 8.22 (d, *J* = 2.0 Hz, 1H), 8.15 (d, *J* = 1.9 Hz, 1H), 7.66 (s, 1H), 2.49 (s, 3H); ^13^C NMR (151 MHz, DMSO) δ 166.3, 148.3, 140.8, 136.1, 135.0, 126.5, 120.0, 21.1.

#### 4.2.2 3‐Methyl‐5‐nitrobenzonitrile (**3**)

At 0°C, a solution of triflic anhydride (11.20 ml, 66.60 mmol) in dichloromethane (50 ml) was added dropwise to a solution of **2** (4.00 g, 22.20 mmol) and triethylamine (15.48 ml, 111.01 mmol) in dichloromethane (150 ml). After a reaction time of 30 min at 0°C, 50 ml of saturated aqueous sodium bicarbonate solution was added, and the mixture was stirred vigorously at room temperature for 10 min. The organic phase was separated, dried over anhydrous MgSO_4_, filtered, and concentrated under reduced pressure. Purification by flash chromatography (80/20 n‐heptane/EtOAc) afforded 3.32 g (92%) of **3** as a white solid. R_*f*_ = 0.55 (80/20 n‐heptane/EtOAc); ^1^H NMR (400 MHz, CDCl_3_) δ 8.33 (td, *J* = 1.5, 0.8 Hz, 1H), 8.28 (dt, *J* = 1.6, 0.8 Hz, 1H), 7.81–7.76 (m, 1H), 2.55 (d, *J* = 0.8 Hz, 3H); ^13^C NMR (101 MHz, CDCl_3_) δ 148.3, 141.8, 138.0, 128.1, 124.4, 116.7, 113.8, 21.2.

#### 4.2.3 3‐Amino‐5‐methylbenzonitrile (**4**)

To a solution of **3** (3.31 g, 20.35 mmol) in MeOH (60 ml) was added iron powder (5.68 g, 101.76 mmol) and glacial acetic acid (11.65 ml, 203.52 mmol). The resulting suspension was stirred under reflux for 2 h. The reaction was cooled to room temperature and water (100 ml) was added. The suspension was filtered on a Celite pad and extracted with EtOAc (3 × 50 ml). The combined organic phase was washed with saturated Na_2_CO_3_ solution (2 × 50 ml), dried over anhydrous MgSO_4_, filtered and concentrated under reduced pressure. Purification by flash chromatography (80/20 n‐heptane/EtOAc) afforded 2.66 g (98%) of **4** as a yellow solid. R_*f*_ = 0.21 (80/20 n‐heptane/EtOAc); ^1^H NMR (400 MHz, CDCl_3_) δ 6.84 (s, 1H), 6.72 (s, 1H), 6.68 (s, 1H), 3.79 (br s, 2H), 2.28 (s, 3H); ^13^C NMR (101 MHz, CDCl_3_) δ 146.8, 140.3, 122.8, 120.0, 119.3, 114.9, 112.8, 21.2.

#### 4.2.4 3‐Iodo‐5‐methylbenzonitrile (**5**)

Concentrated HCl (5 ml) was added to a solution of aniline **4** (2.71 g, 20.43 mmol) in acetone (40 ml) at 0°C. To this mixture was added a chilled solution of sodium nitrite (2.53 g, 36.77 mmol) in water (20 ml), dropwise, with vigorous mechanical stirring. Stirring was continued at 0°C for 15 min after the addition was complete, and then a solution of potassium iodide (6.12 g, 40.86 mmol) in water (20 ml) was added carefully. The cooling bath was removed, and the reaction heated to reflux for 2 h. The mixture was cooled to rt and extracted with DCM (3 × 40 ml). The combined organic extracts were dried (MgSO_4_), filtered, and concentrated under reduced pressure. Purification by flash chromatography (90/10 heptane/EtOAc) afforded 3.8 g (76%) of **5** as a white solid. R_*f*_ = 0.55 (80/20 n‐heptane/EtOAc); ^1^H NMR (400 MHz, CDCl_3_) δ 7.78–7.67 (m, 2H), 7.39 (s, 1H), 2.32 (s, 3H); ^13^C NMR (101 MHz, CDCl_3_) δ 142.6, 141.2, 137.5, 131.8, 117.2, 113.9, 93.9, 20.9.

#### 4.2.5 3‐(Bromomethyl)‐5‐iodobenzonitrile (**6**)

The compound was synthesized as previously described.^6^, ^28^ To a solution of **5** (2.50 g, 10.28 mmol) and *N*‐bromosuccinimide (2.28 g, 12.86 mmol) in MeCN (40 ml) was added AIBN (0.67 g, 4.11 mmol). The reaction was refluxed for 24 h. The solvent was removed under vacuum and the crude purified by flash chromatography (95/5 n‐heptane/EtOAc) to give 1.61 g (49%) of **6** as a white solid. R_*f*_ = 0.28 (95/5 n‐heptane/EtOAc); ^1^H NMR (400 MHz, CDCl_3_) δ 7.96 (d, *J* = 1.6 Hz, 1H), 7.89 (d, *J* = 1.6 Hz, 1H), 7.64 (t, *J* = 1.6 Hz, 1H), 4.38 (s, 2H) ^13^C NMR (101 MHz, CDCl_3_) δ 142.2, 140.9, 140.1, 131.7, 116.6, 114.6, 94.1, 29.9.

#### 4.2.6 Di‐tert‐butyl 2,2′‐((3‐cyano‐5‐iodobenzyl)azanediyl)diacetate (**7**)

The compound was synthesized as previously described.^6^, ^28^ To a solution of **6** (1.00 g, 3.10 mmol) in MeCN (30 ml) was added K_2_CO_3_ (0.64 g, 7.65 mmol) and di‐*tert*‐butyl iminodiacetate (0.91 g, 3.72 mmol). The reaction mixture was stirred at room temperature overnight. The solvent was removed in vacuo, and the resulting mixture was diluted with water (20 ml), extracted with EtOAc (2 × 25 ml), washed with brine (30 ml), dried over MgSO_4_, filtered and concentrated in vacuo. Purification by flash chromatography (90/10 n‐heptane/EtOAc) afforded 1.35 g (89%) of **7** as a colorless oil. R_*f*_ = 0.31 (95/5 n‐heptane/EtOAc); ^1^H NMR (400 MHz, CDCl_3_) δ 7.96 (s, 1H), 7.80 (s, 1H), 7.65 (s, 1H), 3.83 (s, 2H), 3.33 (s, 4H), 1.40 (s, 18H); ^13^C NMR (101 MHz, CDCl_3_) δ 169.9, 142.5, 142.1, 139.3, 131.5, 117.1, 114.1, 93.9, 81.5, 56.2, 55.2, 28.2.

#### 4.2.7 Di‐tert‐butyl 2,2′‐((3‐iodo‐5‐(1,2,4,5‐tetrazin‐3‐yl)benzyl)azanediyl)diacetate (**8**)

The compound was synthesized as previously described.^6^ CH_2_Cl_2_ (0.17 ml, 2.67 mmol), sulfur (0.17 g, 0.67 mmol), hydrazine monohydrate (1.1 ml, 21.39 mmol), and ethanol (4.0 ml) along with **7** (1.31 g, 2.67 mmol) were added to a Biotage microwave vial (10–20 ml) equipped with a stir bar. The vessel was sealed, and the reaction mixture was heated to 50°C for 24 h, before being allowed to cool to room temperature and unsealed. Then 3 ml of CH_2_Cl_2_ and NaNO_2_ (1.84 g, 26.73 mmol) in water (30 ml) were added to the now yellow mixture followed by dropwise addition of acetic acid (10 ml), producing a mixture red in color. The reaction mixture was extracted with CH_2_Cl_2_, washed with brine, dried with MgSO_4_ and filtered before concentrating in vacuo. The crude was purified using flash chromatography (95/5 n‐heptane/EtOAc) to yield 0.37 g (26%) of **8** as a red solid. R_*f*_ = 0.39 (80/20 n‐heptane/EtOAc); ^1^H NMR (600 MHz, CDCl_3_) δ 10.22 (s, 1H), 8.84 (s, 1H), 8.55 (s, 1H), 8.10 (s, 1H), 3.99 (s, 2H), 3.44 (s, 4H), 1.46 (s, 18H); ^13^C NMR (151 MHz, CDCl_3_) δ 170.1, 165.3, 158.0, 142.4, 136.1, 133.4, 129.0, 95.1, 81.4, 56.7, 55.3, 28.2.

#### 4.2.8 2,2′‐((3‐Iodo‐5‐(1,2,4,5‐tetrazin‐3‐yl)benzyl)azanediyl)diacetic acid (**9**)

To a solution of **8** (0.08 g, 0.14 mmol) in CH_2_Cl_2_ (5 ml) was added trifluoroacetic acid (5 ml). The reaction was stirred at room temperature for 2 h. The solvent was then removed under reduced pressure to obtain a red solid. NMR of the crude shows full conversion. Purification by preparative HPLC afforded 0.05 g (62%) of **9** as a pink oil (TFA salt). ^1^H NMR (400 MHz, MeOD) δ 10.30 (s, 1H), 8.87 (s, 1H), 8.64 (s, 1H), 8.14 (s, 1H), 4.45 (s, 2H), 4.01 (s, 4H); ^13^C NMR (101 MHz, MeOD) δ 168.2, 164.7, 158.2, 143.7, 137.7, 134.6, 134.0, 129.6, 94.5, 57.8, 53.6. HRMS (MALDI‐TOF) calculated for C_13_H_13_IN_5_O_4_ [M + H]^+^: 430.0012, found: 430.0014.

#### 4.2.9 Di‐tert‐butyl 2,2′‐((3‐(1,2,4,5‐tetrazin‐3‐yl)‐5‐(trimethylstannyl)benzyl) azanediyl) diacetate (**10**)

The compound was synthesized as previously described.^6^ Pd (PPh_3_)_4_ (19.4 mg, 0.017 mmol) and hexamethylditin (87 μl, 0.42 mmol) were successively added to a microwave vial equipped with a stir bar which was then sealed and purged with N_2_. A solution of **8** (0.08 g, 0.17 mmol) in dry THF (2.5 ml) was added via a syringe and the reaction allowed to stir at 65°C in a microwave for 3 h. The reaction was allowed to cool to room temperature and unsealed before being quenched with saturated aqueous KF (1 ml). The solution was extracted with CH_2_Cl_2_ (3 × 10 ml) washed with brine (10 ml), dried over MgSO_4_, filtered, and concentrated under reduced pressure. The crude was purified using flash chromatography (95/5 n‐heptane/EtOAc) to yield 0.025 g (47%) of **10** as a purple oil. R_*f*_ = 0.37 (80/20 n‐heptane/EtOAc); ^1^H NMR (400 MHz, CDCl_3_) δ 10.20 (s, 1H), 8.63 (d, *J* = 1.8 Hz, 1H), 8.55 (s, 1H), 7.97–7.73 (m, 1H), 4.08 (s, 2H), 3.50 (s, 4H), 1.47 (s, 18H), 0.37 (s, 9H); ^13^C NMR (101 MHz, CDCl_3_) δ 170.1, 166.8, 157.7, 144.3, 141.4, 134.6, 131.1, 129.0, 81.3, 57.4, 55.1, 28.2, −9.3.

### 4.3 Radiochemistry

[^125^I]NaI (>12.95 GBq ml^−1^, 629 GBq mg^−1^) was purchased from Perkin Elmer Life and Analytical Sciences (331 Treble Cove Road, Billerica, MA 01862, USA) as a non‐carrier‐added solution in reductant free 10^−5^ M aqueous sodium hydroxide solution (pH 8–11). Radio‐TLC was carried out on silica gel 60 F_254_ plates or silica gel 60 RP‐18 F2₅₄s from Merck (Germany). Visualization was accomplished by a phosphor imager and analyzed and quantified by OptiQuant. HPLC purification was carried out on a semi‐preparative C18 Luna Column (5 μm, 100 Å, 150 × 10 mm) and monitored with a 254‐nm UV detector and a Raytest Gabi NaI detector. The solvent systems used were water (0.1% TFA, solvent A) and acetonitrile (0.1% TFA, solvent B) with a flow rate of 3 ml/min. Radiochemical conversion (RCC) of all radiolabeled compounds was determined by analyzing a labeled aliquot of the reaction mixture by radio‐TLC and analyzed by integrating the radioactive peaks from the reaction solution. The products were characterized by comparing the R_*f*_ values of the reaction mixture with the R_*f*_ value of the cold reference samples marked on the same TLC plate. The RCY was determined by comparison of the starting activity, which was present in the reaction mixture vial and the radioactivity present in the purified and formulated product. Radioactivity was measured using a dose calibrator and gamma counter.

#### 4.3.1 Radiolabeling of **10**

A modified procedure of Albu et al. was used for radioiodination.^30^ Na[^125^I]I (10 μl, 20 MBq) was added to a solution of stannyl precursor (20 μl from a 5 mg/ml stock in MeOH). Consequently, 1‐μl AcOH was added to the reaction mixture followed by 3‐μl NCS (5 mg/ml) in MeOH. The reaction was agitated for several seconds by hand and left for 10 min. Radio‐TLC (NP, Hex/EA: 4/1, R_*f*_ = 0.52) resulted in an intermediate RCC of 75 ± 12. The crude mixture was subjected to a gentle flow of argon until complete evaporation of the solvent. The crude was redissolved in MeCN and evaporated until dryness, which was repeated three times. A solution of 6‐M HCl_(aq.)_ (200 μl) was added to the crude and left to stir at 60°C. After 2 h, radio‐TLC (RP, MeCN/H_2_O: 1/2) indicated an RCC of 15 ± 11% (*n* = 5). The crude mixture was purified on semipreparative HPLC using a C‐18 column (Luna 5um, C_18_ (2) 100 Å, 250 mm × 10 mm) using a gradient from 15% to 50% B. The desired fraction after purification was added on a Sep‐Pak C18 Plus (Waters) cartridge that was preconditioned by flushing with 10‐ml EtOH followed by 20 ml of H_2_O. The product was eluted with 1 ml of PBS/EtOH (95/5) to a final activity of 1 MBq/ml This corresponds to an RCY of 5%. RCP of 96% was determined with radio‐TLC (SI 2.7).

## CONFLICT OF INTEREST

The authors declare that the research was conducted in the absence of any commercial or financial relationships that could be construed as a potential conflict of interest.

## AUTHOR CONTRIBUTIONS

Conceptualization: MMH, UB, AK, and AIJ. Organic chemistry: UB. Radiochemistry: NB, VS, LH, JTJ, and CBMP. Original draft preparation: NB, UB, and CBMP. Review and editing: NB, UB, CBMP, and MMH. Funding: MMH, AK, and AIJ. All authors read and approved the final manuscript.

## Supporting information


**Data S1.** Supporting Information

## Data Availability

The data are available in the Supporting Information. Moreover, the raw data supporting the conclusions of this article will be made available by the authors, without undue reservation.
