# Supplementary material for: Synthesis and radiolabeling of a polar [125I]I‐1,2,4,5‐tetrazine
Source: J Labelled Comp Radiopharm. 2022 Dec 30;66(1):22–30. doi: 10.1002/jlcr.4009 (PMC10107300; doi:10.1002/jlcr.4009)
Supplement: Supplementary file 1 — Data S1. Supporting Information [file JLCR-66-22-s001.docx]

**Supporting Information**

**Synthesis and Radiolabeling of a polar [^125^I]I-1,2,4,5-Tetrazine**

*Natasha Bidesi^1,†^, Vladimir Shalgunov^1,2,†^, Umberto Maria Battisti^1^, Lars Hvass^3^, Jesper Tranekjær Jørgensen^3^, Christian B. M. Poulie^1^, Andreas I. Jensen^4^, Andreas Kjaer^3^, Matthias M. Herth^1,2,*^*

1. *Department of Drug Design and Pharmacology, Faculty of Health and Medical Sciences, University of Copenhagen, Jagtvej 160, DK-2100 Copenhagen, Denmark.*
2. *Department of Clinical Physiology, Nuclear Medicine and PET, Rigshospitalet, Blegdamsvej 9, 2100, Copenhagen, Denmark*
3. *Cluster for Molecular Imaging, Department of Biomedical Sciences, University of Copenhagen, Blegdamsvej 3, 2100 Copenhagen Ø, Denmark*
4. *Center for Nuclear Technologies (DTU Nutech), Technical University of Denmark (DTU), Frederiksborgvej 399, 4000, Roskilde, Denmark*
5. *Department of Clinical Physiology, Nuclear Medicine and PET & Cluster for Molecular Imaging, Copenhagen University Hospital – Rigshospitalet & Department of Biomedical Sciences, University of Copenhagen, Copenhagen, Denmark*

*^†^ These authors contributed equally to the work*

Contents

[**SI-1 Chemistry:** 4](#_Toc107321813)

[**SI-2 Radio-iodination:** 18](#_Toc107321814)

**SI-1 Chemistry:** *3-Methyl-5-nitrobenzamide (****2****)*

*3-Methyl-5-nitrobenzonitrile (****3****)*

*3-Amino-5-methylbenzonitrile (****4****)*

*3-Iodo-5-methylbenzonitrile (****5****)*

*3-(Bromomethyl)-5-iodobenzonitrile (****6****)*

*Di-tert-butyl 2,2'-((3-cyano-5-iodobenzyl)azanediyl)diacetate* *(****7****)*

*Di-tert-butyl 2,2'-((3-iodo-5-(1,2,4,5-tetrazin-3-yl)benzyl)azanediyl)diacetate (****8****)*

*2,2'-((3-Iodo-5-(1,2,4,5-tetrazin-3-yl)benzyl)azanediyl)diacetic acid (****9****)*

’

*Di-tert-butyl 2,2'-((3-(1,2,4,5-tetrazin-3-yl)-5-(trimethylstannyl)benzyl)azanediyl) diacetate (****10****)*

# **SI-2 Radio-iodination:**

**SI-2.1 Preparative HPLC purification Radio-chromatogram of [^125^I]I-Tz 12**


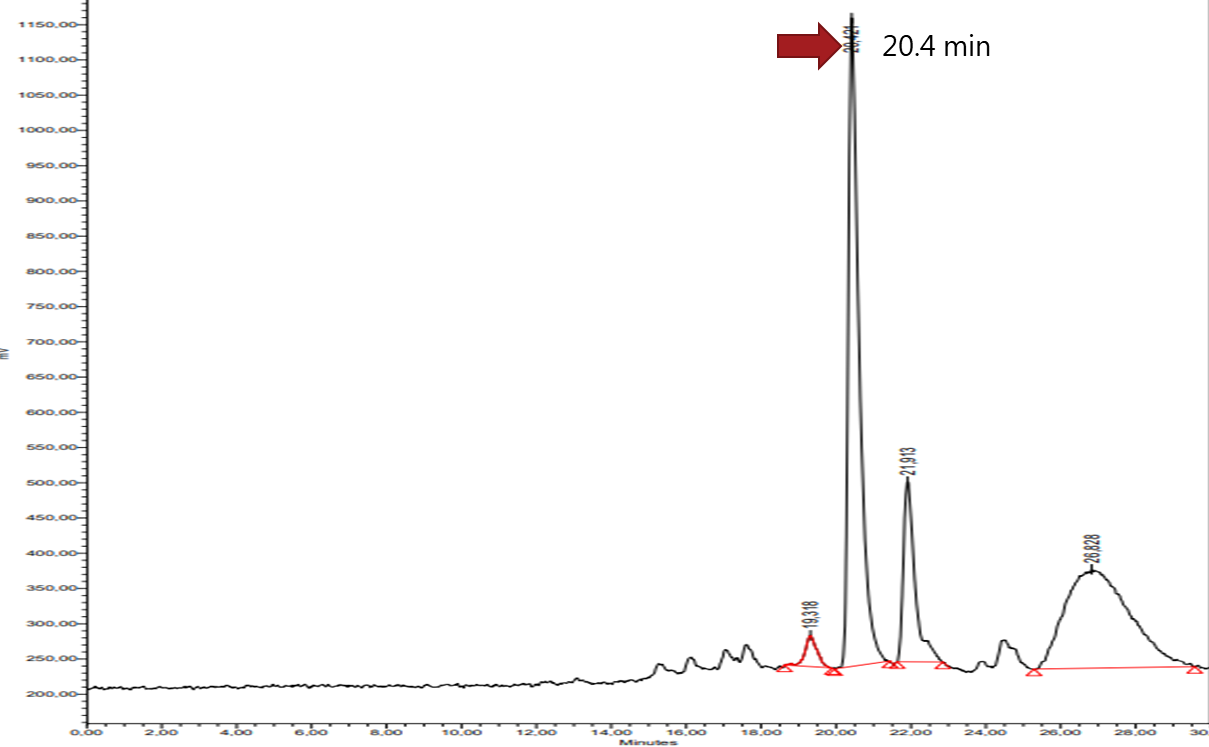


Collected product peak is indicated with an arrow.

**SI-2.2 Preparative HPLC purification UV-chromatogram [^125^I]I-Tz 12**

**
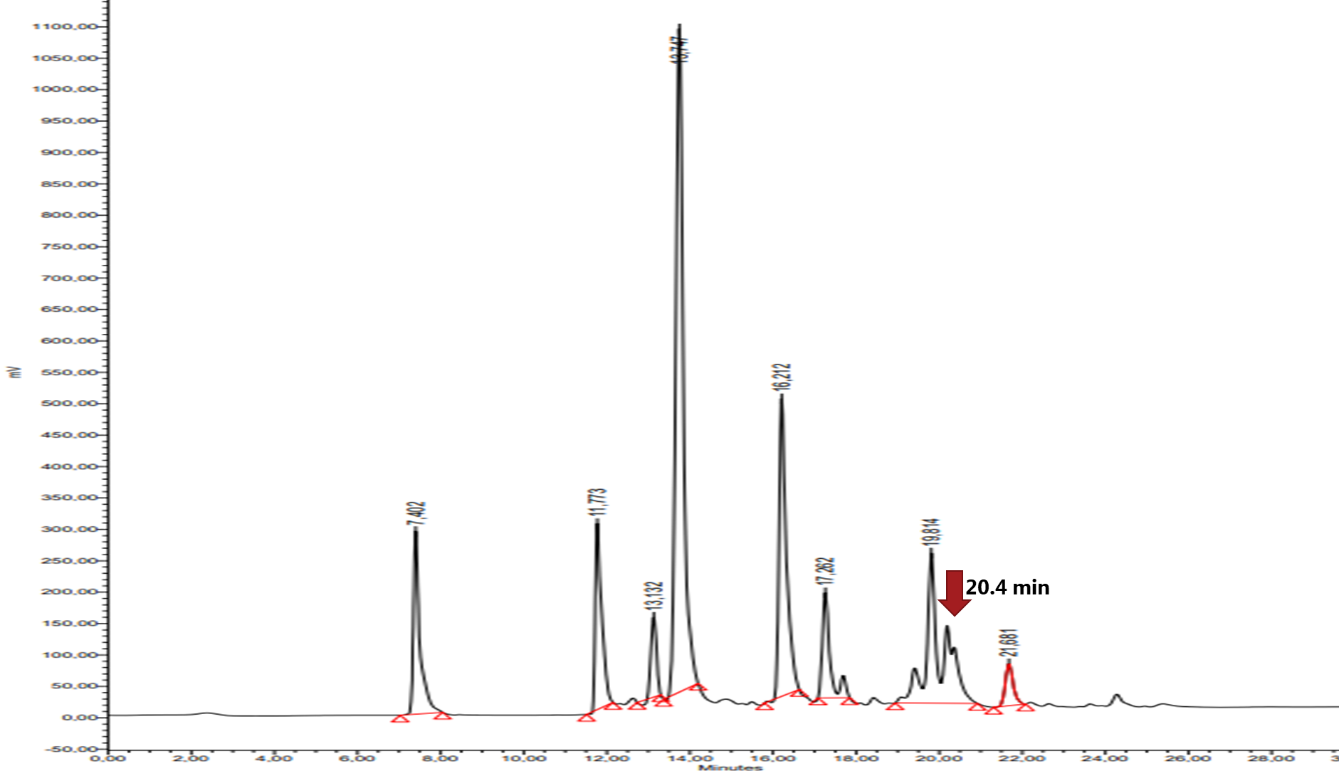
**

**SI-2.3 [^125^I]I-Tz 11 Radio-TLC labeling**


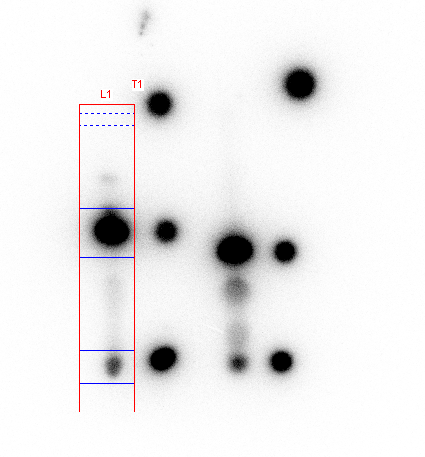
**
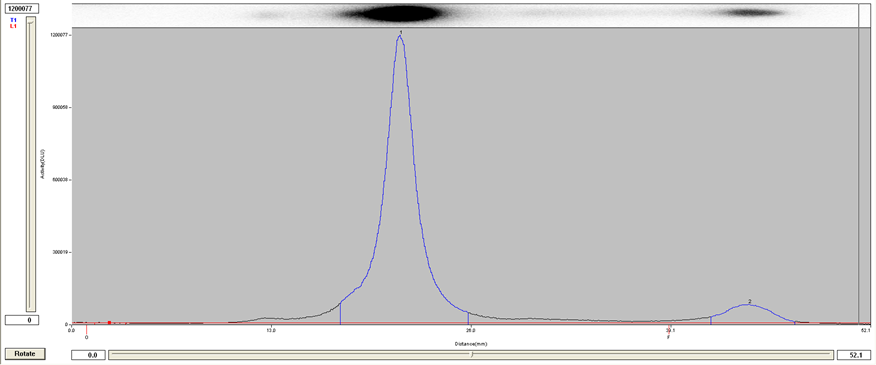
**

NP-TLC (Hex:EA = 4:1); RCC = 75%

Right Lane: **[^125^I]I-Tz 11 crude** : R_f_: 0.6

Left Lane: R_f_: 0, R_f_: 1 and cold reference marked with ^125^I

**SI-2.4 [^125^I]I-Tz 12 crude radio-TLC after 2.5 h deprotection**


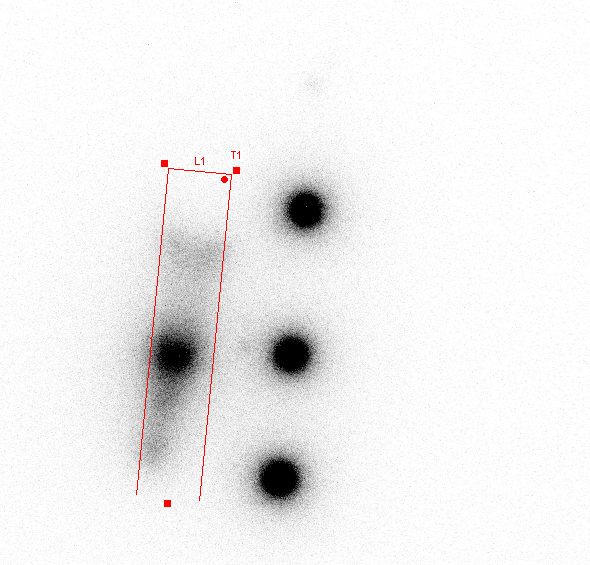

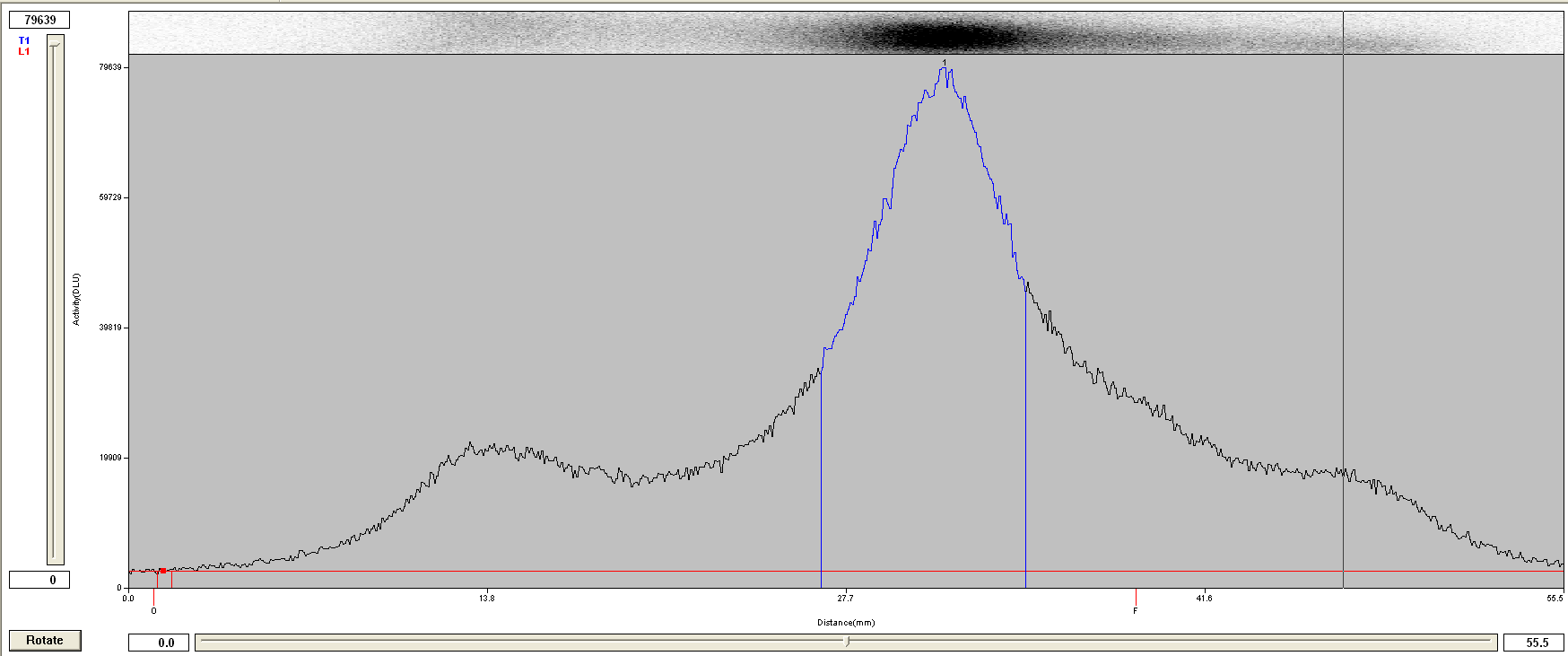


RP-TLC (MeCN:H_2_O = 1:2), RCC of 38%

Right Lane: **[^125^I]I-Tz 12 crude** : R_f_: 0.4

Left Lane: R_f_: 0, R_f_: 1 and cold reference marked with ^125^I

**SI-2.4 Radio-TLC after preparative HPLC purification of [^125^I]I-Tz 12**


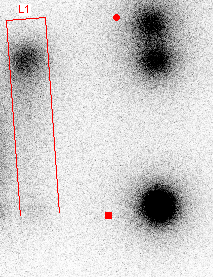
 **
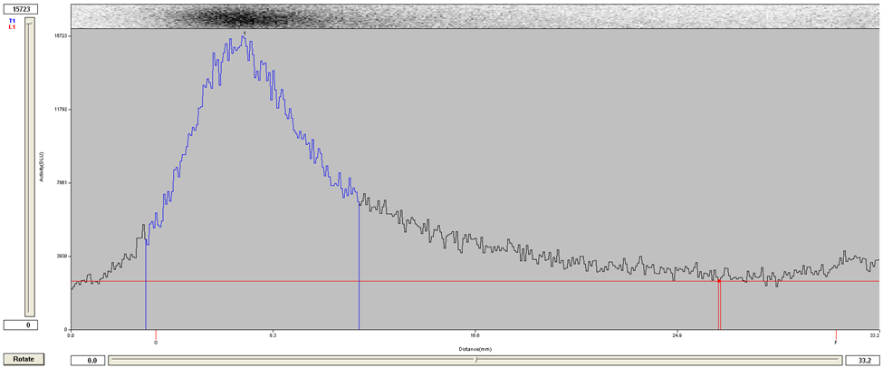
**

RP-TLC (MeCN:H_2_O = 1:1)

Right Lane: **[^125^I]I-Tz 12** after preparative HPLC purification: R_f_: 0.8. RCP:80%

Left Lane: R_f_: 0, R_f_: and cold reference marked with ^125^I

**SI-2.7 [^125^I]I-Tz 17 Radio-TLC stability**


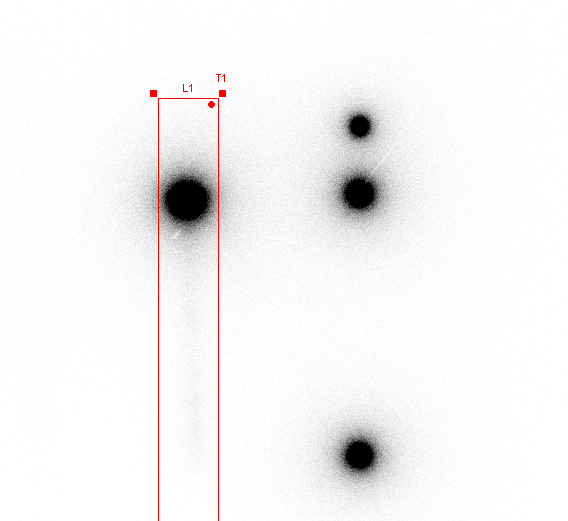

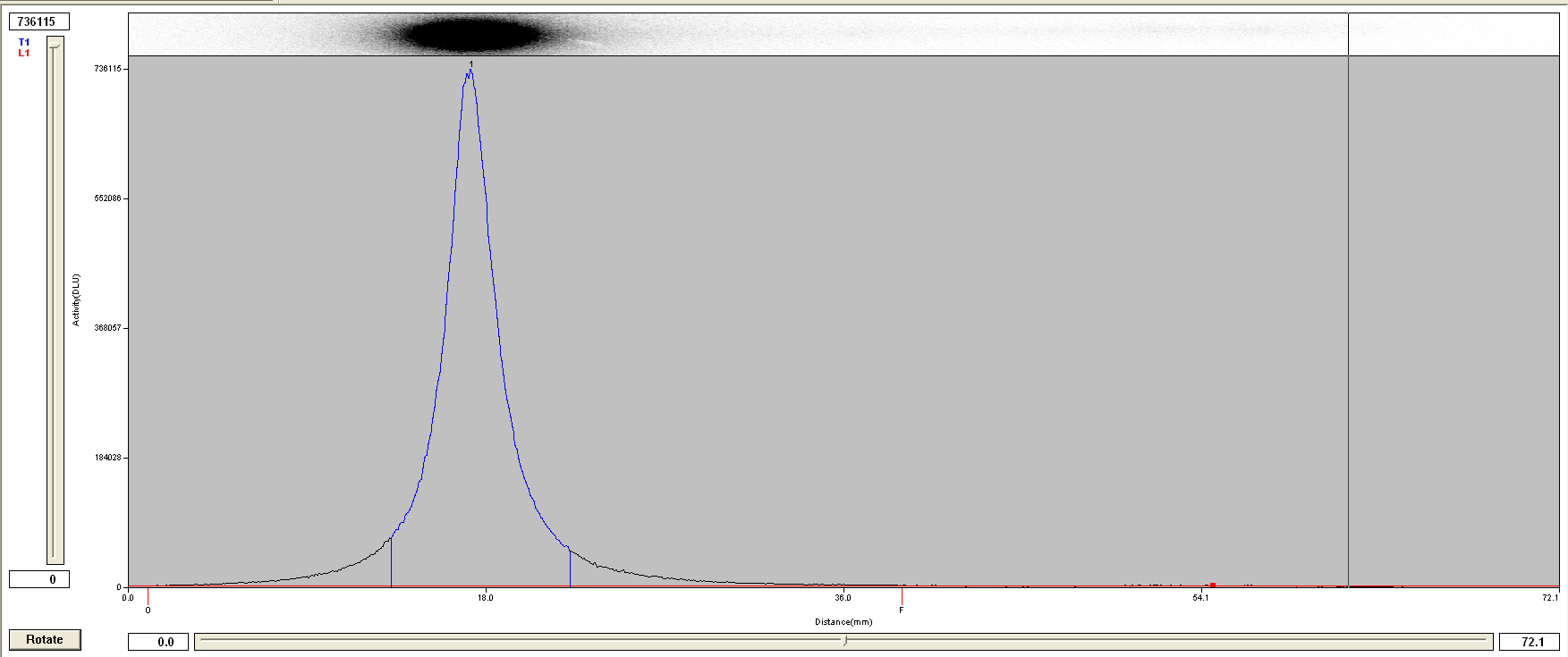


RP-TLC (MeCN:H_2_O = 1:1)

Right Lane: **[^125^I]I-Tz 12 after 24h in 95/5 PBS/EtOH**: R_f_: 0.8, RCP: 97%

Left Lane: R_f_: 0, R_f_: 1 and cold reference marked with ^125^I

**SI-2.8 [^125^I]I-Tz 12 ligation with TCO-PNB**

Reaction procedure:

100 uL of a 1 mg/mL mixture of TCO-PNB (excess) was added to 1 uL of the final product (1 MBq/mL) 24h and 48h after formulation and run on radio-TLC (RP-TLC (MeCN:H_2_O = 1:2). The reference was prepared by adding the cold reference of tetrazine **12** to TCO-PNB ester in a 1:2 equivalence (spotted on lane 3), and this reference spot was marked by NaI.


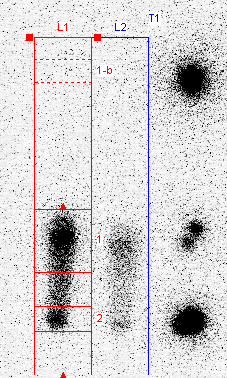

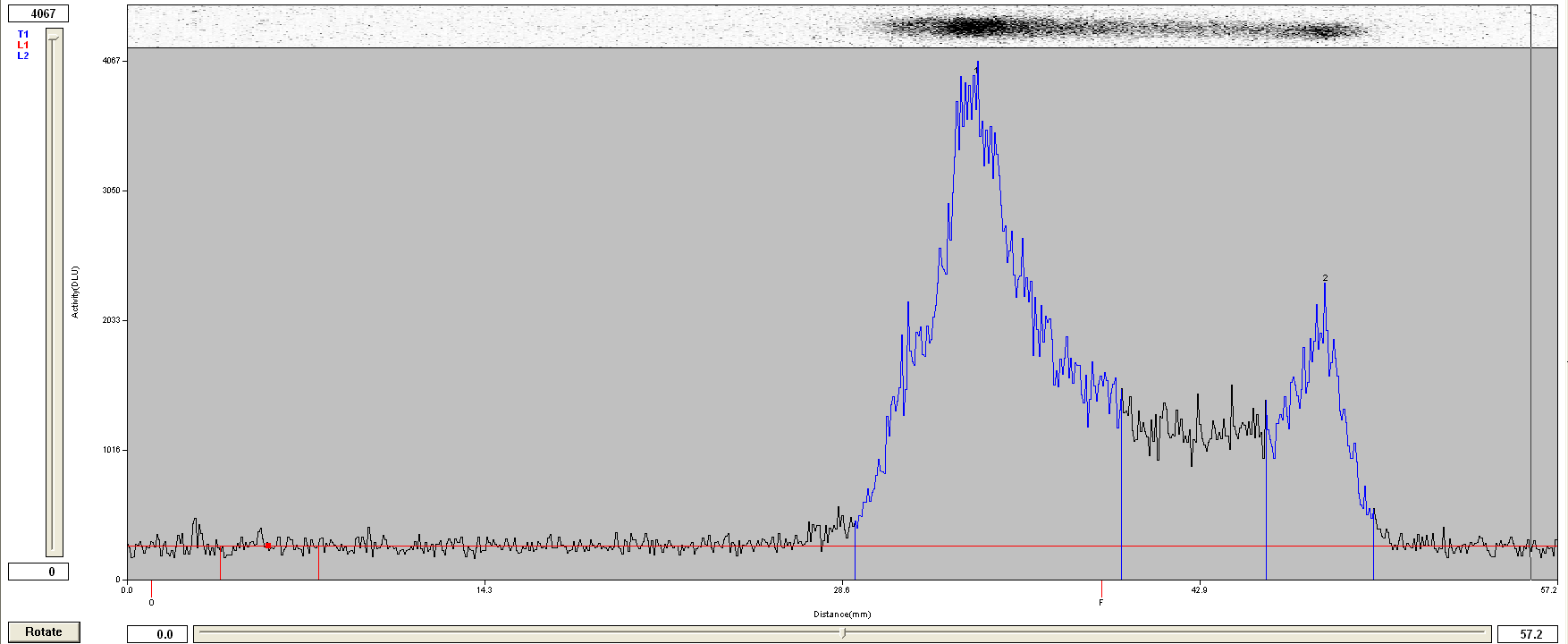


RP-TLC (MeCN:H_2_O = 1:1)

Right Lane: **[^125^I]I-Tz 12** ligation with **TCO-PNB** after 24h: R_f_: 0.4

Middle Lane: **[^125^I]I-Tz 12** ligation with **TCO-PNB** after 48h: R_f_: 0.4

Left Lane: R_f_: 0, R_f_: 1 and cold reference of clicked products marked with ^125^I
